# Supplementary material for: Transition approaches for autistic young adults: A case series study
Source: PLoS One. 2022 May 5;17(5):e0267942. doi: 10.1371/journal.pone.0267942 (PMC9070902; doi:10.1371/journal.pone.0267942)
Supplement: S1 File — (DOCX) [file pone.0267942.s001.docx]

**Category 1: Improvement in Quality of Life**

The following three cases are presented to further examine potential risk and protective factors associated with successful transition to adulthood or improved QoL.

**Case 1 - Ms. Katherine**

***Demographic Characteristics*.** Ms. Katherine is a 20-year-old single female, studying at TAFE, receives the disability pension, and lives with a roommate. Her daily activities include doing the household chores and seeking employment. Ms. Katherine received a diagnosis of ASD at the age of 19. She therefore has had her diagnosis for a period of 1 year.

***Education, Support, and Intervention****.* Ms. Katherine attended a state high school and completed year 12*.* At school, Ms. Katherine did not receive a transition plan, but completed three work experience placements, and received social skills training. Thus, Ms. Katherine completed her school education, and is engaged in post-secondary education to obtain specific skills to enhance her transition.

***Quality of Life*.** Ms. Katherine showed clinically reliable improvement for total QoL, Satisfaction, Competence/Productivity, Empowerment/Independence and Social Belonging scores from baseline to follow-up, thus indicating that overall, she is satisfied with her life situation and experiencing an improvement in her QOL. RCI scores for total QoL and subscales from baseline to follow-up are presented in Table 1.

Table 1

*Summary of QoLQ, RCI, and MSPSS Categories for Ms. Katherine*

|  | T1 | T2 | RCI | Category |  | T1 | T2 | T1 | T2 |
| --- | --- | --- | --- | --- | --- | --- | --- | --- | --- |
| QOLT | 63 | 87 | +19.09 | Reliable improvement | MSPSST | 3 | 3 | Moderate | Moderate |
| SAT | 13 | 18 | +3.97 | Reliable improvement | SSO | 4 | 1 | Moderate | Low |
| CP | 11 | 24 | +10.34 | Reliable improvement | SF | 2 | 1 | Low | Low |
| EI | 22 | 25 | +2.38 | Reliable improvement | SFr | 3 | 6 | Moderate | High |
| SB | 17 | 20 | +2.38 | Reliable improvement |  |  |  |  |  |

*Note*. Quality of Life Questionnaire (QoLQ) and subscale abbreviations, Satisfaction (SAT), Competence/Productivity (CP), Empowerment/Independence (EI), and Social Belonging (SB). Multidimensional Scale of Perceived Social Support Total (MSPSST) and subscale abbreviations, Support from Significant Other (SSO), Support from Family (SF), and Support from Friends (SFr).

MSPSS scores from 1 to 2.9 indicate low support, 3 to 5 moderate support, and 5.1 to 7 high support (Zimet et al., 1988).

***Social Support***. Perceived social support scores show an increase on the Support from Friends subscale from baseline to follow-up for Ms. Katherine. At baseline, in her words, Ms. Katherine reported that her friends, “*kinda bully me”*. However, at follow-up Ms. Katherine described her relationship with her friends as *“amazing, they are my everything”*. Thus, Ms. Katherine’s relationships with her friends appeared to have improved over time.

With regards to relationships, perceived social support showed a decrease in the Support from a Significant Other subscale. In her words at baseline, Ms. Katherine described her life as, “*lonely, due to being single”*. In addition, Ms. Katherine reported at baseline that, “*I rarely interact with others and usually stuff up on social cues*”. Thus, it would appear that Ms. Katherine experienced difficulty socialising and also missed having support from a special person in her life. Perceived social support from family on the Support from Family subscale remained low for Ms. Katherine, whilst the total Multidimensional Scale of Perceived Social Support score remained moderate. Overall, it might be the case that social support from Ms. Katherine’s friends could be a buffer in the absence of support from family and a significant person in her life.

***Challenges***. At baseline, low scores on the Competence/Productivity subscale and the Support from Family subscale, indicated challenges associated with access to employment, and family support. In particular, Ms. Katherine reported two major challenges, first in finding employment, and second, in her relationship with her family. Specifically, Ms. Katherine associated the negative impact of her ASD in social and communication challenges, with limited access to job opportunities, and in her words at baseline, she reported that she was, “*disappointed as I can’t find a job and getting nowhere to my goals.*” Further, both at baseline and follow-up, Ms. Katherine reported a difficult relationship with her family as indicated in consistently low scores on the support from family subscale. Specifically, she does not communicate with her mother, and reported, *“We don’t talk due to different points of view on how I should live*.” Thus, this challenge in relationships with her mother was reflected in consistently low scores on the Support from Family subscale at baseline and at follow-up.

***Positive Experiences***. At follow-up, scores on the Competence/Productivity, Empowerment/Independence, and Social Belonging subscales showed clinically reliable improvement for Ms. Katherine, thus indicating overall improvement in access to employment, independence in daily living, and increased community integration. Notably, over the 12-month transition period, Ms. Katherine reported being happy as she gained part-time employment as a waitress, and reported earning wages of $100 per week. Thus, Ms. Katherine viewed gaining employment, earning a wage, and integrating with the community as positive experiences at follow-up.

***ASD Impact***. At follow-up, Ms. Katherine reported a positive attitude towards her ASD. In her words, “*I know who I am now and am a happier, bubblier person*.” Thus, it would appear that Ms. Katherine’s late diagnosis at the age of 19 gave her a sense of identity in understanding her unique self, and may have contributed to her improvement in QoL over time.

**Case 2 - Ms. Talita**

***Demographic Characteristics***. Ms. Talita is a 22-year-old female who lives with her partner and does not receive the disability pension. Ms. Talita is engaged in post-secondary education, is in full-time employment in an administrative role for eighteen months, and receives a wage of over $200 per week. Ms. Talita received a diagnosis of ASD at age 21. She therefore has had her diagnosis for a period of 1 year.

***Education, Support, and Intervention***. Ms. Talita attended a private high school and completed year 12. However, Ms. Talita did not receive social support or life skills interventions at school. In addition, she did not access work experience placements and was not on a transition plan. Thus, it is likely that as Ms Talita received a late diagnosis post high school, within the Australian school disability support system, she was not eligible for support, interventions, and transition planning at high school (Disability Programs, 2017).

***Quality of Life****.* Ms. Talita showed clinically reliable improvement in scores on the total QOL, Satisfaction, and Competence/Productivity subscale scores. Scores on the Empowerment/Independence remained high over the 12-month transition period, whilst Social Belonging remained low and showed no change. RCI scores for total QoL and subscales from baseline to follow-up are presented in Table 2.

Table 2

*Summary of QoLQ, RCI, and MSPSS Categories for Ms. Talita*

|  | T1 | T2 | RCI | Category |  | T1 | T2 | T1 | T2 |
| --- | --- | --- | --- | --- | --- | --- | --- | --- | --- |
| QOLT | 79 | 99 | +15.91 | Reliable improvement | MSPSST | 5 | 5 | Moderate | Moderate |
| SAT | 15 | 25 | +7.95 | Reliable improvement | SSO | 7 | 7 | High | High |
| CP | 21 | 28 | +5.57 | Reliable improvement | SF | 4 | 4 | Moderate | Moderate |
| EI | 23 | 25 | +1.59 | No reliable change | SFr | 4 | 3 | Moderate | Moderate |
| SB | 20 | 21 | +0.79 | No reliable change |  |  |  |  |  |

*Note*. Quality of Life Questionnaire (QoLQ) and subscale abbreviations, Satisfaction (SAT), Competence/Productivity (CP), Empowerment/Independence (EI), and Social Belonging (SB). Multidimensional Scale of Perceived Social Support Total (MSPSST) and subscale abbreviations, Support from Significant Other (SSO), Support from Family (SF), and Support from Friends (SFr).

MSPSS scores from 1 to 2.9 indicate low support, 3 to 5 moderate support, and 5.1 to 7 high support (Zimet et al., 1988).

***Social Support***. Perceived social support remained high on the Support from Significant Other subscale, and moderate on the total Multidimensional Scale of Perceived Social Support score, Support from Family, and Support from Friends subscales for Ms. Talita. As reported, Ms. Talita required regular assistance from her partner in time management and in organising her daily morning routine that was important to her, as she stated, “*he is my everything”*. Ms. Talita reported having some friends in her previous hometown, however in her new place of living she reported that she does, “*try to get involved sometimes*.” Thus, it would appear that Ms. Talita is dependent on support from her partner and this support is important in her successful transition in daily living activities and functional independence.

***Challenges*.** Ms. Talita reported no major challenges, however, she did note some difficulty in levels of stress and anxiety she experienced at baseline, as her employer had high expectations of her in her administrative employment role. However, at follow-up, whilst Ms. Talita reported still feeling stressed, in her own words she noted, “*but overall things are good*.” Thus, it would appear that Ms. Talita was able to manage this stress and had adjusted well to her new place of employment.

***Positive Experiences***. Increased scores on the Competence/Productivity subscale score over time indicated improved access to employment opportunities for Ms. Talita that may have assisted in her successful transition. In particular, over the follow-up period, Ms. Talita reported feeling competent and productive in her new occupation and stated that she,” *feels like she is making a difference”*. This was a change from her first assessment at baseline, when Ms. Talita reported being stressed and anxious at her place of employment, in her words, “*there are lots of expectations by the higher up bosses*”. During the 12-month transition period, Ms. Talita moved interstate, got married, started a new administrative job, all of which she identified as changes that were positive experiences.

***ASD Impact***. For Ms. Talita, whilst she reported improvement in QoL, there were still notable ways in which her ASD impacted her daily life. Overall, she reported ongoing difficulty socialising, in time management, and daily organisation that continued to present challenges for her.

**Case 3 - Ms. Petal**

***Demographic Characteristics****.* Ms. Petal is a 20-year-old single female who lives with her parents. She attends university and receives the disability pension. Her daily activity consists of surfing the web, engaging in online chat rooms, and reading about history. Ms. Petal received an ASD diagnosis at the age of 5. She therefore has had her diagnosis for a period of 15 years.

***Education, Support, and Intervention***. Ms. Petal attended a state high school and received support and intervention through the special education program. At school, Ms. Petal received a transition plan with parent involvement in transition planning, and received the following interventions: behaviour support, social skills training, and life skills training. Additionally, Ms. Petal completed three work experience placements whilst at high school. Ms. Petal reported that she received social support, and life skills training post high school from her family.

***Quality of Life****.*  Ms. Petal showed clinically reliable improvement in scores on the total QOL, Satisfaction, Empowerment/Independent, and Social Belonging subscales, with scores remaining low on the Competence/Productivity subscale over time. RCI scores for total QoL and subscales from baseline to follow-up are presented in Table 3.

Table 3

*Summary of QOLQ, RCI, and MSPSS Categories for Ms. Petal*

|  | T1 | T2 | RCI | Category |  | T1 | T2 | T1 | T2 |
| --- | --- | --- | --- | --- | --- | --- | --- | --- | --- |
| QOLT | 72 | 82 | +7.95 | Reliable improvement | MSPSST | 5 | 6 | Moderate | High |
| SAT | 15 | 18 | +2.38 | Reliable improvement | SSO | 4 | 7 | Moderate | High |
| CP | 19 | 18 | -0.79 | No reliable change | SF | 6 | 6 | High | High |
| EI | 21 | 25 | +3.18 | Reliable improvement | SFr | 5 | 5 | Moderate | Moderate |
| SB | 17 | 21 | +3.18 | Reliable improvement |  |  |  |  |  |

*Note*. Quality of Life Questionnaire (QoLQ) and subscale abbreviations, Satisfaction (SAT), Competence/Productivity (CP), Empowerment/Independence (EI), and Social Belonging (SB). Multidimensional Scale of Perceived Social Support Total (MSPSST) and subscale abbreviations, Support from Significant Other (SSO), Support from Family (SF), and Support from Friends (SFr).

MSPSS scores from 1 to 2.9 indicate low support, 3 to 5 moderate support, and 5.1 to 7 high support (Zimet et al., 1988).

***Social Support***. Perceived social support scores show an increase on the total Multidimensional Scale of Perceived Social Support score, and Support from Significant Other, whilst Support from Family remained high, and Support from Friends remained moderate for Ms. Petal. In particular, Ms. Petal reported a close relationship with her mother and brother as she stated, “*amazing, especially my mum, and my brother is my main companion*.” She also reported having one friend online, who understood her autism. In her words Ms. Petal reported, “*I’m in charge if I want to socialise or not*”, indicating that Ms. Petal most likely chose times to socialise with her friend in an attempt to manage her friendship. Thus, the close relationship with her family and the support of her friend were important to Ms. Petal.

***Challenges*.** Low scores on the Satisfaction, Competence/Productivity, and Social Belonging subscales at baseline indicated that Ms. Petal was unhappy with her overall situation, and had difficulty accessing employment. In particular, Ms. Petal reported being unhappy with her living circumstances, being unemployed, and had difficulty integrating with the community, as she reported in her own words she, “*barely leaves the house”.* Further, Ms. Petal reported that she would prefer to live independently and be in employment. Interestingly, an increase in scores on the Satisfaction, Empowerment/Independence, and Social Belonging subscales was noted at follow-up. Specifically, at follow-up, Ms. Petal reported that she was,” *able to go out shopping, and to the doctor by herself, and am somewhat fulfilled*.” Thus, it would appear that an increase in functional independence in daily activities improved community integration and satisfaction for Ms. Petal, which was important to her overall wellbeing.

***Positive Experiences***. The only positive experience reported by Ms. Petal was having her friend. As reported earlier, support from her friend was important to Ms. Petal.

***ASD Impact***. For Ms. Petal, whilst she reported improvement in QoL, and having a friend, there were still notable ways in which her ASD impacted her daily life. Overall, Ms. Petal reported difficulty in making new friends, being lonely, and experienced challenges in, “*being able to socialise properly”.* Thus*,* it would appear that social and communication challenges associated with ASD had a negative impact on Ms. Petal and her ability to make friends.

**Category 2: Deterioration in Quality of Life**

The following three cases are presented to further examine potential risk and protective factors associated with unsuccessful transition or deterioration in QoL.

**Case 1 - Ms. Lavender**

***Demographic Characteristics*.** Ms. Lavender is a 19-year-old single female, lives with her parents, and does not receive the disability pension. She is unemployed, not engaged in post-secondary education, and describes her daily activity as staying home with her parents. Ms. Lavender is a volunteer for one day a week in a disability support service. Ms. Lavender experiences anxiety and depression, and received an ASD diagnosis at the age of 4. She therefore has had her diagnosis for a period of 15 years.

***Education, Support, and Intervention.*** Ms. Lavender attended a state high school and completed year 12. At high school, Ms. Lavender received a transition plan with parental involvement in transition planning, received social skills training, and completed three work experience placements.

***Quality of Life.*** Ms. Lavender showed clinically reliable deterioration in scores on the total QOL, Satisfaction, and Social Belonging subscales from baseline to follow-up, whilst Competence/Productivity subscale scores remained low over time. Empowerment/Independent subscale scores remained low over time indicating that Ms. Lavender experienced difficulty in functional independence in daily life activities. RCI scores for total QoL and subscales from baseline to follow-up are presented in Table 4.

Table 4

*Summary of QoLQ, RCI, and MSPSS Categories for Ms. Lavender*

|  | T1 | T2 | RCI | Category |  | T1 | T2 | T1 | T2 |
| --- | --- | --- | --- | --- | --- | --- | --- | --- | --- |
| QOLT | 75 | 64 | -8.75 | Reliable deterioration | MSPSST | 3 | 3 | Moderate | Moderate |
| SAT | 20 | 13 | -5.57 | Reliable deterioration | SSO | 1 | 1 | Low | Low |
| CP | 20 | 20 | 0 | No reliable change | SF | 4 | 4 | Moderate | Moderate |
| EI | 17 | 16 | -0.79 | No reliable change | SFr | 4 | 3 | Moderate | Moderate |
| SB | 18 | 15 | -2.38 | Reliable deterioration |  |  |  |  |  |

*Note*. Quality of Life Questionnaire (QOLQ) and subscale abbreviations, Satisfaction (SAT), Competence/Productivity (CP), Empowerment/Independence (EI), and Social Belonging (SB). Multidimensional Scale of Perceived Social Support Total (MSPSST) and subscale abbreviations, Support from Significant Other (SSO), Support from Family (SF), and Support from Friends (SFr).

MSPSS scores from 1 to 2.9 indicate low support, 3 to 5 moderate support, and 5.1 to 7 high support (Zimet et al., 1988).

***Social Support*.** Perceived social support scores remained moderate on the total Multidimensional Scale of Perceived Social Support score, Support from Family, and Support from Friends subscales over time, whilst Support from a Significant Other remained low for Ms. Lavender throughout the transition period. Although Ms. Lavender reported being close to her mother, in her words, her mother was, “*angry and disappointed about the fact that I am still unemployed*.” Further, she hoped that her involvement with her friends could be better and reported that, “*I adore my friends dearly, but I don't want to burden them”.* With regards to a partner, Ms. Lavender reported that she was, “*unfortunately single*.” Thus, it would appear that whilst her social support scores remained moderate, Ms. Lavender would like to be more engaged with her friends, and missed the support from a special person in her life. Further, whilst she was close to her mother, she felt as though she was disappointing her by being unemployed, which impacted this relationship.

***Challenges*.** Low scores on the Satisfaction and Social Belonging subscales indicated that overall, Ms. Lavender was unhappy with her life situation and experienced difficulty integrating with the community. Indeed, in her words, Ms. Lavender reported, “*When it comes to the community I am not very sociable due to my anxiety. A few years ago, it was a struggle to get me to go to the supermarket.*” Further, over the 12-month transition period, Ms. Lavender reported that she was disappointed with her life, felt unsuccessful, and continued to experience anxiety and depression. Thus, Ms. Lavender experienced additional challenges in socialising, and managing her anxiety and depression that appears to have impacted her overall transition.

***Positive Experiences*.** Ms. Lavender was unable to identify any positive experiences over the 12-month follow-up period.

***ASD Impact*.** For Ms. Lavender, the impact of her ASD and associated challenges appeared to have a considerable effect in her daily life. According to Ms. Lavender, she reported difficulty in integrating with the community and in socialising with her friends. In her words, Ms. Lavender reported, “*I have anxiety and depression as well and that factors into my emotions and lifestyle*. *I can understand why I think and live in a different way because of my diagnosis.*” Overall, although Ms. Lavender was cognisant of her differences and appeared to embrace them in a positive light, her ASD challenges appeared to continue to impact her in major areas of her life.

**Case 2 - Ms. Kelly**

***Demographic Characteristics.*** Ms. Kelly is a 21-year-old female who lives with roommates in shared accommodation. She is in a relationship, does not receive the disability pension, is unemployed, and is not engaged in post-secondary education. Ms. Kelly experiences anxiety and received an ASD diagnosis at the age of 13. She therefore has had her diagnosis for a period of 8 years.

***Education, Support, and Intervention*.** Ms. Kelly attended a private high school and completed Year 12. At school, Ms. Kelly did not receive support or interventions and did not access work experience placements. She did not receive a transition plan, social skills, or life skills training.

***Quality of Life*.** Ms. Kelly showed clinically reliable improvement in scores on the Competence/Productivity subscale, whilst scores on the total QoL, Satisfaction, and Social Belonging subscales showed clinically reliable deterioration from baseline to follow-up, and scores on the Empowerment/Independence subscale remained low over time. RCI scores for total QoL and subscales from baseline to follow-up are presented in Table 5.

Table 5

*Summary of QoLQ, RCI, and MSPSS Categories for Ms. Kelly*

|  | T1 | T2 | RCI | Category |  | T1 | T2 | T1 | T2 |
| --- | --- | --- | --- | --- | --- | --- | --- | --- | --- |
| QOLT | 69 | 66 | -2.38 | Reliable deterioration | MSSPSST | 4 | 6 | Moderate | High |
| SAT | 25 | 19 | -4.77 | Reliable deterioration | SSO | 4 | 7 | Moderate | High |
| CP | 10 | 15 | +3.98 | Reliable improvement | SF | 4 | 5 | Moderate | Moderate |
| EI | 12 | 13 | +0.79 | No reliable change | SFr | 4 | 4 | Moderate | Moderate |
| SB | 22 | 19 | -2.38 | Reliable deterioration |  |  |  |  |  |

*Note*. Quality of Life Questionnaire (QoLQ) and subscale abbreviations, Satisfaction (SAT), Competence/Productivity (CP), Empowerment/Independence (EI), and Social Belonging (SB). Multidimensional Scale of Perceived Social Support Total (MSPSST) and subscale abbreviations, Support from Significant Other (SSO), Support from Family (SF), and Support from Friends (SFr).

MSPSS scores from 1 to 2.9 indicate low support, 3 to 5 moderate support, and 5.1 to 7 high support (Zimet et al., 1988).

***Social Support*.** Perceived social support scores showed an increase on the total Multidimensional Scale of Perceived Social Support score and Support from Significant Other, whilst Support from Family, and Support from Friends for Ms. Kelly remained moderate over time. With respect to support from friendships, Ms. Kelly noted specific concerns that appeared to influence her functioning over the follow-up period. For example, she reported difficulty maintaining friendships due to her anxiety and in her words, she reported, “*I’m a bit of a hermit. My anxiety can prevent me from actively keeping friendships*.” However, Ms. Kelly did report that she was in a long-distance relationship and found comfort and support in her partner. Thus, social support remained reasonable for Ms. Kelly over the transition period.

***Challenges*.** Low scores on the Satisfaction and Social Belonging subscales indicated that, overall Ms. Kelly was unhappy with her life situation, and experienced difficulty integrating with the community during her transition process. Ms. Kelly reported it particularly problematic that she rarely participated in any social or community activities and that she subsequently was often lonely. Further, in her words, she reported that she was, “*anxious, I feel my life is useless, less successful than others*”. Thus, Ms. Kelly’s challenges in coping with anxiety and difficulty with social interactions appeared to impact quite substantially on her life situation, overall wellbeing, and happiness.

***Positive Experiences*.** Interestingly, clinically reliable improvement in scores on the Competence/Productivity subscale was noted at follow-up, evident through a change in access to employment for Ms. Kelly. Specifically, over the 12-month transition period, Ms. Kelly moved interstate, lived independently, gained part-time employment in a childcare setting, and reported earning between $100 to $150 a week. More importantly, at follow-up, Ms. Kelly reported feeling happy, and that she had made an impact in the child-care setting. In her words she stated, “*I help them learn and grow. It makes me really happy to know I’m making an impact even if it’s a small one*”. Thus, it would appear that gaining employment, earning a wage, and contributing meaningfully to the community improved Ms. Kelly’s feelings of competence and productivity, which was important to her. However, Ms. Kelly lived independently in her new living circumstances, and, whilst this may be positive, it may present additional challenges for her in functional independence as she is working minimal hours. It is therefore likely that improvement in her productivity in employment did not transfer into her overall wellbeing, perhaps due to the additional difficulties experienced with social support, anxiety, friendships, and community integration.

***ASD Impact*.** Overall, whilst Ms. Kelly was aware of the challenges associated with her ASD, she appeared to embrace them from a positive perspective. In her words Ms. Kelly reported, “*My diagnosis means that I know what’s going on with my body and brain. I think being autistic also makes me more sympathetic to the struggles of others, but there is still some stigma attached*”. Thus, she demonstrates acceptance and understanding, yet identifies that she still experiences stigma associated with ASD. This may continue to contribute to her overall QOL.

**Case 3 - Ms. Kylie**

***Demographic Characteristics.*** Ms. Kylie is a 25-year-old single female who lives with a roommate in shared accommodation and receives the disability pension. She is unemployed and not engaged in post-secondary education. Ms. Kylie reported requiring prompting from her roommates in organising herself in daily activities on a regular basis. Her daily activity consists of staying home with roommates, playing video games, and watching documentaries. Ms. Kylie received a diagnosis of ASD at the age of 19. She therefore has had her diagnosis for a period of 6 years.

***Education, Support, and Intervention*.** Ms. Kylie attended a private high school and completed Year 12. At school, Ms. Kylie did not receive support or interventions, and did not access any work experience placements. She did not receive a transition plan. Thus, it is likely that as Ms Kylie received a late diagnosis post high school, within the Australian school disability support system she was not eligible for support, interventions, and transition planning at high school.

***Quality of Life****.* Ms. Kylie showed clinically reliable deterioration in scores for the total QOL and Satisfaction subscales, whilst scores on the Social Belonging scale remained high from baseline to follow-up. Scores on the Competence/Productivity and Empowerment/Independence subscale remained low for Ms. Kylie over time, thus indicating that Ms. Kylie had difficulty with skills, experience, and access to employment, as well as difficulty with functional independence in daily life activities. Notably at baseline, Ms. Kylie reported high QOL most likely due to high levels of satisfaction and social belonging at baseline RCI scores for total QOL and subscales from baseline to follow-up are presented in Table 6.

Table 6

*Summary of QoLQ, RCI, and MSPSS Categories for Ms. Kylie*

|  | T1 | T2 | RCI | Category |  | T1 | T2 | T1 | T2 |
| --- | --- | --- | --- | --- | --- | --- | --- | --- | --- |
| QOLT | 85 | 78 | -5.57 | Reliable deterioration | MSSPSST | 4 | 5 | Moderate | Moderate |
| SAT | 27 | 24 | -2.38 | Reliable deterioration | SSO | 4 | 6 | Moderate | High |
| CP | 15 | 14 | -0.79 | No reliable change | SF | 5 | 4 | Moderate | Moderate |
| EI | 18 | 17 | -0.79 | No reliable change | SFr | 4 | 4 | Moderate | Moderate |
| SB | 25 | 23 | -1.59 | No reliable change |  |  |  |  |  |

*Note*. Quality of Life Questionnaire (QoLQ) and subscale abbreviations, Satisfaction (SAT), Competence/Productivity (CP), Empowerment/Independence (EI), and Social Belonging (SB). Multidimensional Scale of Perceived Social Support Total (MSPSST) and subscale abbreviations, Support from Significant Other (SSO), Support from Family (SF), and Support from Friends (SFr).

MSPSS scores from 1 to 2.9 indicate low support, 3 to 5 moderate support, and 5.1 to 7 high support (Zimet et al., 1988).

***Social Support*.** Ms. Kylie reported an increase in perceived social support over the transition period as evident on the Support from Significant Other subscale, whilst scores remained moderate over time for the total Multidimensional Scale of Perceived Social Support, Support from Family, and Support from Friends subscales for Ms. Kylie. Whilst Support from Friends remained moderate, Ms. Kylie reported having little involvement with friends apart from her roommate. In her words, “*I am* *completely content having very little involvement with people. I don’t have a drive to be social”.* Further, Ms. Kylie reported talking to her parents occasionally, and believed that they, *“still treat me like a child*”. Overall, although social support scores were moderate, support appeared to be limited for Ms. Kylie, yet this did not cause her distress.

***Challenges*.** A decrease in scores on the Satisfaction subscale indicated that Ms. Kylie was unhappy with her life situation over the transition period. In particular, Ms. Kylie reported sensory struggles that impacted her friendships. In her words, she stated that she was, *“considered fussy*, *not knowing how friendships work, struggling to find the balance between being a pushover and being aggressive.”* Further, low scores in Empowerment/Independence over time, indicated that Ms. Kylie experienced difficulty in functional independence in daily living skills. More specifically, Ms. Kylie reported that she was able to do the bare minimum on a daily basis, in her words, she reported, “*feeling frustrated and disappointed”*. Thus, a lack of independence in daily living skills and difficulty socialising presented challenges for Ms. Kylie.

***Positive Experiences*.** Despite some struggles with social support, Ms Kylie reported making “*solid friends”* through her online gaming community and feels valued for her contributions to a variety of online networks. She noted this as a particularly positive experience for her indicating the importance of online social interactions for Ms. Kylie.

***ASD Impact*.** For Ms. Kylie, the impacts of an ASD and associated challenges appeared to have a considerable effect on her daily life activities. Ms. Kylie acknowledged her understanding of difficulties associated with her ASD and appeared to be accepting of them. Further, she was able to clearly articulate her self-regulation strategies in managing her ASD challenges through a positive lens. With respect to her ASD, Ms. Kylie reported, “*I actually have an understanding of why I struggle with certain things rather than just thinking I'm a bad or defective person. I also know that if I'm unreasonably angry, I'm probably having a sensory issue and can try to do something about it*”. Overall however, Ms. Kylie continued to experience difficulty in daily organisation and struggled to understand how friendships worked.

**Category 3: No Change in Quality of Life**

The following case is presented to further examine potential risk and protective factors associated with successful and unsuccessful transition to adulthood.

**Case 1 - Ms. Lily**

***Demographic Characteristics****.* Ms. Lily is a single 19-year old female who attends university, lives with her parents, and receives the disability pension. Ms. Lily received an ASD diagnosis at the age of 12. She therefore has had her diagnosis for a period of 7 years

***Education, Support, and Intervention****.* Ms. Lily attended a state high school and completed Year 12. At school Ms. Lily received a transition plan with parental involvement in transition planning, had one work experience placement, and received both social skills, and life skills training.

***Quality of Life*.** Ms. Lily showed no reliable change in total QoL scores from baseline to follow-up, although her subscale profile was more complicated. However, reliable improvement was shown for scores on the Competence/Productivity subscales, whilst scores on the Satisfaction and Social Belonging subscales showed reliable deterioration over time for Ms. Lily. Scores on the Empowerment/Independence subscale remained high over time. RCI scores for total QoL and subscales from baseline to follow-up are presented in Table 7.

Table 7

*Summary of QoLQ, RCI, and MSPSS Categories for Ms. Lily*

|  | T1 | T2 | RCI | Category |  | T1 | T2 | T1 | T2 |
| --- | --- | --- | --- | --- | --- | --- | --- | --- | --- |
| QOLT | 79 | 80 | 0.79 | No reliable change | MSPSST | 3 | 3 | Moderate | Moderate |
| SAT | 20 | 16 | -3.18 | Reliable deterioration | SSO | 2 | 2 | Low | Low |
| CP | 12 | 23 | +8.75 | Reliable improvement | SF | 2 | 2 | Low | Low |
| EI | 23 | 23 | 0 | No reliable change | SFr | 4 | 4 | Moderate | Moderate |
| SB | 24 | 17 | -5.57 | Reliable deterioration |  |  |  |  |  |

*Note*. Quality of Life Questionnaire (QOLQ) and subscale abbreviations, Satisfaction (SAT), Competence/Productivity (CP), Empowerment/Independence (EI), and Social Belonging (SB). Multidimensional Scale of Perceived Social Support Total (MSPSST) and subscale abbreviations, Support from Significant Other (SSO), Support from Family (SF), and Support from Friends (SFr).

MSPSS scores from 1 to 2.9 indicate low support, 3 to 5 moderate support, and 5.1 to 7 high support (Zimet et al., 1988).

***Social Support***. Perceived social support scores remained moderate for the total Multidimensional Scale of Perceived Social Support, and Support from Friends subscales, whilst scores on the Support from Significant Other, and Support from Family subscales remained low for Ms. Lily. Ms. Lily reported recently moving interstate, and that she did not have any new friendships. In her words, she stated, “*The only friends I have are in another state, and they have autism like me, so we really understand each other and get along really well”.* Ms. Lily’s inability to have formed new friendships in her new location appeared to be an important factor in limiting her successful transition. However, as her move interstate was only recent, it is likely that building social relationships will improve as she makes new friends, which might impact her overall QoL. Further, Ms. Lily also noted difficulty in relationships with her family, which may have impacted her transition. In her words, she reported, *“My family has started to distance themselves from me, as now I'm older they think I should be grown out of my autism*”. Thus, it would appear that Ms. Lily experienced limited support from her family, most likely due to their misunderstanding of her ASD. It is likely that this may have limited her ability to successfully transition during this period.

***Challenges***. Low scores on the Satisfaction and Social Belonging subscales indicated that overall, Ms. Lily was unhappy with her life situation, and experienced difficulty integrating with the community. In particular, Ms. Lily reported challenges with social inclusion after having disclosed her ASD at university. More specifically, she reported that, “*I came out about my autism at university last year when I was doing a community services course, and my trainer said I should have hid my autism from her and not told them. Then I was excluded from group activity through the whole course”.* Thus, Ms. Lily clearly experienced challenges navigating the university sector associated with stigma relating to her diagnosis. Ultimately, she appeared to be negatively impacted by being socially excluded in this activity whilst at university. In addition, Ms. Lily reported missing her friends in her home town and in her words, she stated, “*When I say I have autism, there are people who back away from me immediately, so I struggle a lot with making friends*”. Thus, it would appear that Ms. Lily experienced several negative experiences associated with stigma and ASD, which ultimately impacted her ability to make friends and engage productively at university.

***Positive Experiences***. Ms. Lily reported an increase in scores on the Competence/Productivity subscale at follow-up. Of note, over the 12-month transition period, Ms. Lily moved interstate, and was a volunteer at an autism day-care centre working with young children on the autism spectrum. In her words, Ms. Lily reported, “*It just makes me happy helping kids out that are going through the same thing, not knowing how to interact with others or understand humour*”. Thus, Ms. Lily reported feeling competent and productive, with increased self-esteem, through this volunteer experience. It is possible that this may have at least in part buffered the effects of the challenges reported by Ms. Lily.

***ASD Impact***. Ms. Lily demonstrated a positive attitude towards her ASD, as she reported in her words, “*I love spreading awareness about autism and talking about it because I see autism as a part of me not something wrong with me”.* Thus, whilst Ms. Lily was aware of the challenges associated with her ASD in her daily life, she appeared to embrace them from a positive perspective as part of her unique identity.
