# Supplementary material for: Transition approaches for autistic young adults: A case series study
Source: PLoS One. 2022 May 5;17(5):e0267942. doi: 10.1371/journal.pone.0267942 (PMC9070902; doi:10.1371/journal.pone.0267942)
Supplement: Attachment — Submitted filename: Response to Reviewers (2).docx [file pone.0267942.s002.docx]

**Manuscript** [PONE-D-21-38878] - [EMID:d9095635f8aecf81]

**Response to Reviewers**

Dear A/Professor Webster and Reviewers

Thank you for giving us the opportunity to submit a revised version of the manuscript “Transition Approaches for autistic young adults: a case series study” for publication in Plos One. We appreciate the time and effort that you and the reviewers have dedicated to providing feedback and are grateful for the insightful comments and suggestions for valuable improvements to our paper. We have incorporated all of the suggestions made by the reviewers. Those changes are highlighted within the manuscript and described below. Omissions referred to by Reviewer 1 are addressed in table format with reviewer comment and author responses sequentially. In addition, please see below, in table format, for a point-by-point response to both the editor and reviewers’ comments and concerns. All page numbers refer to the revised manuscript file.

Kind regards

Yosheen Pillay

| Editor Comment | Author Response |
| --- | --- |
| Please provide additional details regarding participant consent | Detailed information regarding informed consent can be found on page 13 under Procedure and has been revised to enhance clarity. |
| Please upload a copy of Supporting Information S1 which you refer to in your text on pages 8, 15 and 28. | This has been included and uploaded with the revised manuscript |
| Please review your reference list to ensure that it is complete and correct. | This has been checked and updated in accordance with the revised manuscript |

| **Reviewer 1 Comment** | **Author Response** |
| --- | --- |
| Mr Reggie-Consider whether his mental health issues could be the reason for his difficulties socialising and finding employment | Thank you for this suggestion. We believe this is an important consideration and have edited as such. A sentence has been added to reflect this on page 27 |
| **Reviewer 2 Comment** | **Author Response** |
| What ASD Diagnosis did each participant receive and how many years have participants had their diagnosis | This information has been included on page 7. Additional information regarding age of diagnosis, length, and type of diagnosis (e.g. ASD, aspergers) has been included in each of the cases in the supplementary file submitted as an attachment as well as the two cases in the text. |
| Please provide a copy of the *About You* *Survey* instrument | We are pleased to share this author developed *About You Survey*. This has been included in the submission |
| Table 1 correction of typo to add up to 9 participants under Program | Apologies for this typo. This has been corrected in Table 1 under Program to reflect a total of 9 participants |
| Limitation - access to computer was a limitation to only participants who had a computer | Thank you for highlighting this important point. We acknowledge this as a limitation and have now included this as part of our limitation section on P33. As suggested, we have also suggested that multiple approaches to data collection would have potentially increased the sample size. |
| **Reviewer 3 Comment** | **Author Response** |
| There is limited reference to the transition literature in general and for young adults with autism | We agree and have reviewed the literature and included additional commentary within the introduction as well as 10 new references to reflect the richness of transitions in general and specifically for autistic individuals. This is now also included in the reference list. |
| The richness of the quality of life literature is missing | We agree and have reviewed the literature and included additional commentary within the introduction as well as 7 new references to reflect the richness of transitions in general and specifically for autistic individuals. This is now also included in the reference list. |
| Strengths and weaknesses of a case series design | We have edited the study aims section on pages 5 and 6 to temper the description of a case series design and focus on strengths and weaknesses of this methodological approach |
| **Results** |  |
| The demographic results are clearly presented but there is no discussion of them in the text. | Thank you for highlighting this omission. We have now added a discussion the demographic and individual factors in the results section on page 15. |
| Is there a reason for the overwhelming number of female participants? | We acknowledge your thoughtful comments relating to gender. It certainly was interesting in this study to see our sample represented by 7 women and only 2 men, and we thank you for highlighting the need to discuss this in the paper.  We have added in detailed information regarding the gender split at the time of data collection both at Time 1 and Time 2 which can be seen in Figure 1 and on page 12. At Time 1 the initial participant response was from 10 males and 6 females. Three males were excluded due to being outside the age of the inclusion criteria. At Time 2 only 10 participants responded with 3 males lost to attrition and one male excluded due to an incomplete dataset. Thus, the final sample for analysis comprised of 7 females and 2 males. |
| Page 7 Line 163  The word significant needs to be replaced in this sentence as the design does not allow for statements such as this to be made. | Thank you for highlighting this claim in our results section. We acknowledge and have removed the word significant on page 7. |
| Line 173  Other measures that in line 173 they state as being indicators of successful and unsuccessful transitions. | This sentence has been tempered to include the word ‘potential’ on page 7. |
| Can two open ended questions added to the About Me survey really assess changes in quality of life | The Quality of Life Questionnaire was used to measure QoL as per the questions in the different domains. The addition of two open ended questions allowed participants to provide additional information regarding their experiences over the past twelve months. This also allowed participants who wished to explain their QoL experiences in more depth. These were not used to measure QoL, rather to inform case descriptions and identification of factors related to the positive experiences and challenges faced during this transition period. We have amended the description in text on page 9. |
| Provide support for cut scores of 79 and 80 | There are no published clinical cut-offs for the QoLQ, however, according to the Standardisation Manual (Schalock & Keith, 2004) individuals with disability in semi-independent or independent living, engaged in employment, with increased community integration, with a total QoL score of 80 and above are identified as having a high QoL. Those individuals with disability in supervised accommodation, unemployed, with low levels of satisfaction, and a total QoL score of 79 and below are identified as having a low QoL (Schalock & Keith, 2004).  In order to examine differences during the transition period for participants who showed deterioration and improvement in levels of QoL, in consultation with the instrument authors, a median split of the sample based on total QoL scores was conducted at follow-up (K. Keith, R. Schalock, personal communication, 16 June 2017). Thus, based on the Standardisation Manual (Schalock & Keith, 2004), high QoL was defined as a total QoL score of 80 and above, and low QoL was defined as a total QoL score of 79 and below. We have provided this additional information on page 10 of the text under the Quality of Life questionnaire. |
| Reanalyse the results using subscale scores of the Quality of Life questionnaire | We agree that an analysis of the subscales would provide a comprehensive presentation the transition experience and quality of life outcomes in the different domains. As such, we have completed a reanalysis and included our findings on pages 19, 20 and 21. |
